# A High-Reliability Piezoelectric Tile Transducer for Converting Bridge Vibration to Electrical Energy for Smart Transportation

**DOI:** 10.3390/mi14051058

**Published:** 2023-05-17

**Authors:** Thanh Huyen Pham, Thanh Danh Bui, Toan Thanh Dao

**Affiliations:** 1 Faculty of Electrical-Electronic Engineering, University of Transport and Communications, No. 3 Cau Giay Street, Hanoi 100000, Vietnam; 2 Faculty of Mechanical Engineering, University of Transport and Communications, No. 3 Cau Giay Street, Hanoi 100000, Vietnam

**Keywords:** flexible piezoelectric, piezoelectric tile transducer, electronic reliability, energy for smart transportation

## Abstract

Piezoelectric energy transducers offer great potential for converting the vibrations of pedestrian footsteps or cars moving on a bridge or road into electricity. However, existing piezoelectric energy-harvesting transducers are limited by their poor durability. In this paper, to enhance this durability, a piezoelectric energy transducer with a flexible piezoelectric sensor is fabricated in a tile protype with indirect touch points and a protective spring. The electrical output of the proposed transducer is examined as a function of pressure, frequency, displacement, and load resistance. The maximum output voltage and maximum output power obtained were 6.8 V and 4.5 mW, respectively, at a pressure of 70 kPa, a displacement of 2.5 mm, and a load resistance of 15 kΩ. The designed structure limits the risk of destroying the piezoelectric sensor during operation. The harvesting tile transducer can work properly even after 1000 cycles. Furthermore, to demonstrate its practical applications, the tile was placed on the floor of an overpass and a walking tunnel. Consequently, it was observed that the electrical energy harvested from the pedestrian footsteps could power an LED light fixture. The findings suggest that the proposed tile offers promise with respect to harvesting energy produced during transportation.

## 1. Introduction

Electrical energy harvested from ambient sources is critical in intelligent transport systems for addressing the problems posed by climate change and global warming [1,2]. Renewable energy technologies for transportation can be divided into three main approaches: solar radiation harvesting, heat harvesting, and mechanical energy harvesting. Among them, mechanical energy harvesting based on the piezoelectric effect is an important method since electrical energy can be directly obtained from transportation operations. Using the vibrational forces from cars moving on a bridge or road or by harnessing human footsteps on the street, a piezoelectric transducer produces an electric charge, which can provide a power source for traffic control, structural health monitoring, internet-of-things devices, electric vehicles, or lighting on the street [3,4,5,6,7,8,9,10,11,12,13,14]. 

Structurally, a piezoelectric energy-harvesting transducer consists of a piezoelectric active layer sandwiched between a couple of electrodes. Lead zirconate titanate (PZT) nanofibers are widely used in the active layer because they exhibit a strong piezoelectric response and mechanical flexibility [2], which are conducive to the creation of a high-performance device. So far, most studies on piezoelectric harvesting transducers using PZT nanofibers for transportation have focused on enhancing power levels [5,6,7,8,9,10]. Reliability is an important factor of an electronic system that must be investigated. Recently, several groups have mentioned this factor [5,6,7,10]. For example, Ammar et al. [5] used a piezoelectric sensor composed of PZT in an energy-harvesting shoe. This device is able to harvest energy at low frequencies and through irregular chock-like footstep input excitation. However, the degradation of the PZT sensor occurs rapidly in this process, limiting the operation of the harvester to only a few cycles [5]. He et al. [6] fabricated a piezoelectric-energy-harvesting floor structure. An amplification mechanism was designed to enhance the force produced by a pedestrian. The maximum peak-to-peak voltage increased from 18.8 V to 51.4 V, but these results were paired with a relatively poor stability of 40 operating cycles. On the other hand, an inspiring concept was reported by Ahn et al. [7], who designed a bending-type piezoelectric structure with a displacement-amplifying mechanism. Although this design helped to significantly limit the risk of destroying the piezoelectric sensor, there were only 110 operating cycles recorded in the harvesting transducer device. Overall, to bring piezoelectric harvesting transducers closer to implementation in practical applications, it is necessary to perform additional research to enhance the stability property [4].

In this paper, we introduce a high-stability energy-harvesting tile designed and fabricated based on a flexible PZT nanofiber piezoelectric sensor. Dependences of output voltage and output power on applied pressure, load resistance, displacement, and frequency are experimentally investigated. Thanks to the flexibility of the sensor and a mechanical design with an indirect touch point and a protection spring, our harvesting transducer tile can work properly even after 1000 cycles.

## 2. Flexible Piezoelectric Sensor

**Figure 1:**
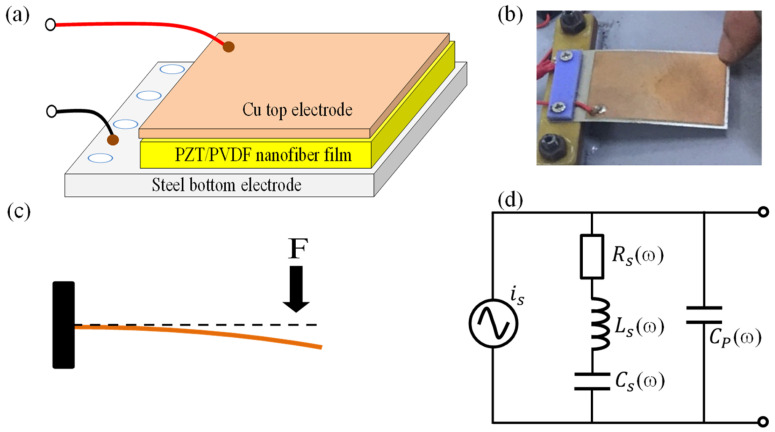
(**a**) Device structure of sensor; (**b**) photo of flexible piezoelectric sensor; (**c**) illustration of the operation of the sensor; (**d**) equivalent circuit of sensor.

A 50 mm × 35 mm × 0.2 mm piezoelectric sensor (provided by PZT Electronic, China) was employed to construct the piezoelectric energy transducer reported in this study. The piezoelectric sensor was fabricated based on the nanofibers of a poly vinylidene fluoride (PVDF) polymer and PZT composites sandwiched between a steel bottom electrode and a Cu thin-film top electrode (Figure 1a). The sensor’s flexibility helps enhance the mechanical-to-electrical and endurance characteristics of the energy transducer tile. Regarding the sensor’s operation, when external pressure is applied to the free part of the flexible piezoelectric sensor (Figure 1b,c), it is elastically deformed. This deformation results in a flow of electric charge. The output electrical signal of the piezoelectric sensor in response to an applied mechanical force was tested, the results of which are presented in Video S1. It is well known that a vibration energy transducer requires a frequency-matching procedure that renders the natural frequency of the piezoelectric sensor equal to the frequency of the input force [9,10]. Unfortunately, the manufacturer does not provide the corresponding parameters; thus, they needed to be determined before the sensor could be used to fabricate the harvesting transducer tile. We reused the model reported by H. Jabbar et al. [10], as shown in Figure 1d.

The impedance (*Z*) of the sensor’s equivalent circuit is complex, consisting of a reactive and real part, as presented in the following equations:

(1) $$\;Z\left(\omega \right)=R_{s}\left(\omega \right)-jX_{s}\left(\omega \right).$$

(2) $$\left|Z\left(\omega \right)\right|=\sqrt{R_{s}^{2}\left(\omega \right)+X_{s}^{2}\left(\omega \right).}\;$$

**Figure 2:**
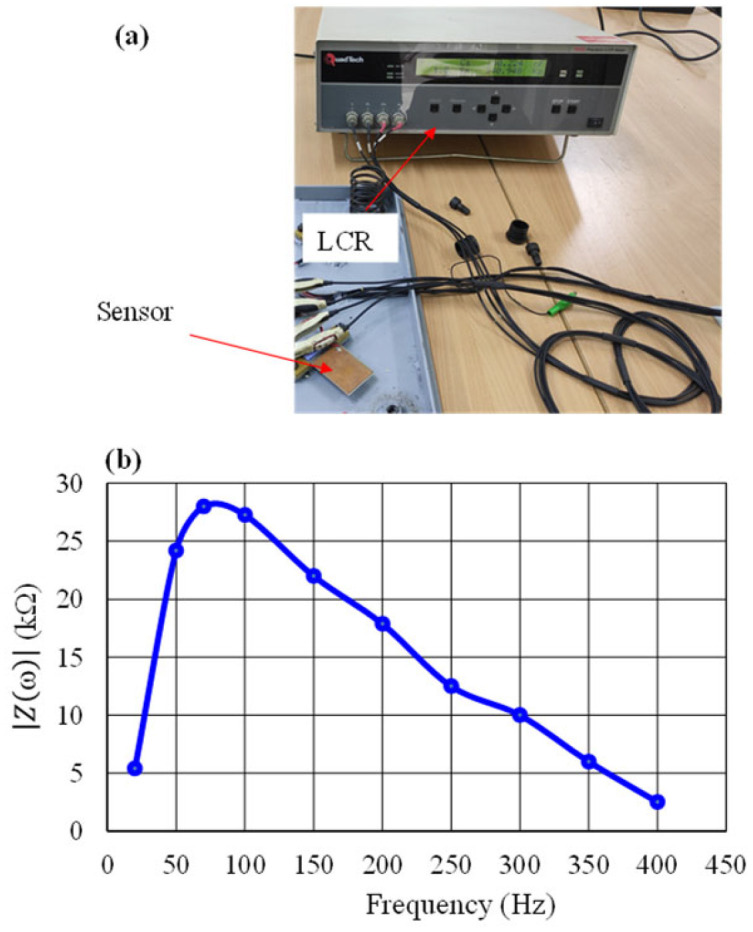
(**a**) Photo of impedance measurement of piezoelectric sensor. (**b**) Frequency dependence of magnitude with real and imaginary parts measured using LCR.

We performed an additional experiment on the impedance measurement using QuadTech LCR Meters, as shown in Figure 2a. The modulus of *Z* was calculated with Equation (2) using the measured values of the reactive and real part at the frequency from 20 Hz to 400 Hz. As can be seen in Figure 2b and Table 1, the resonant frequency is about 75 Hz, which will be used in our further experiments (shown in Section 4). The *Z* modulus values in Table 1, ranging from 2.52 kΩ to 28.2 kΩ, are relatively high. However, they are similar to those reported in previous work [10]. 

## 3. Piezoelectric Energy-Harvesting Transducer

**Figure 3:**
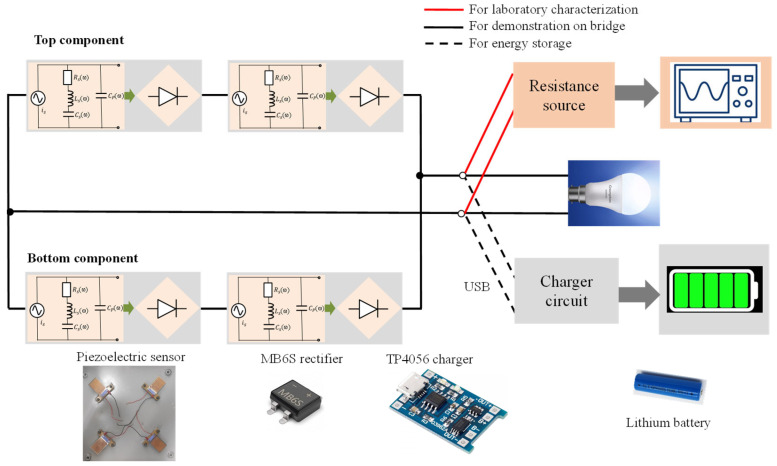
Electronic circuit diagram of energy transducer.

The electronic circuit diagram of the energy transducer is shown in Figure 3. In order to double the current and voltage, a structure of four piezoelectric sensor panels was designed. Two components were connected in parallel; meanwhile, in each component, a pair of sensors was connected in series. Here, we have not utilized many sensors due to the problems of parasitic capacitance and device–device capacitance occurring during the device’s operation, which cause low responsivity [5,6,7,8,9,10,11,12,13,14]. A rectifier circuit using a four-diode bridge device (MB6S, Fairchild, WI, USA) was attached to convert AC values to DC values. A TP4056 (ICSTORE, Delhi, India) charging circuit was used to connect a lithium battery and a rectifier. 

**Figure 4:**
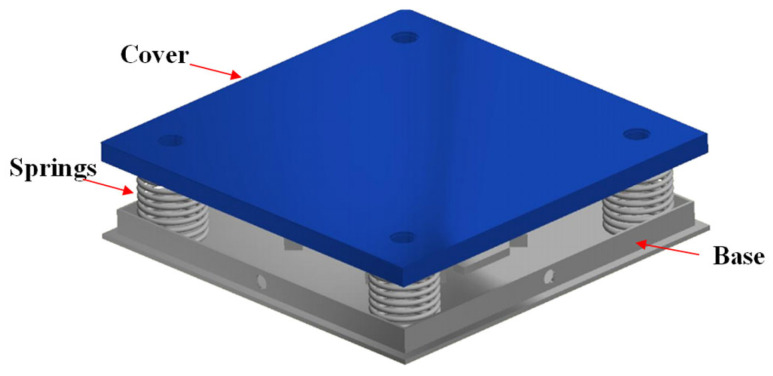
Piezoelectric energy-harvesting transducer designed in 3D using SolidWorks 2022.

For the device’s mechanical design, we used SolidWorks 2022 to render the harvesting transducer tile, as shown in Figure 4. The harvesting transducer was designed with dimensions of 400 mm × 400 mm × 70 mm, which are equal to those in tiles widely used for bridge floor construction.

**Figure 5:**
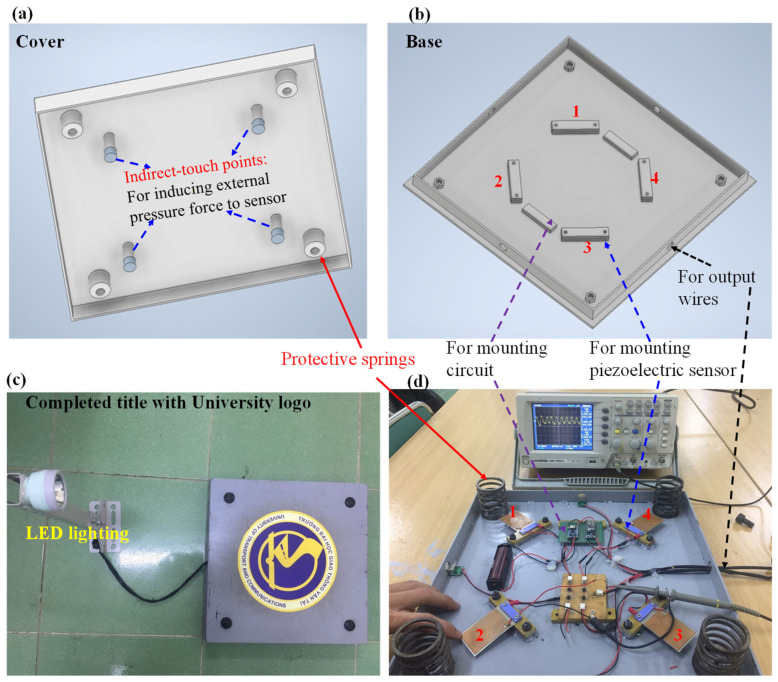
Diagrams of (**a**) cover and (**b**) base and photos of overall view (**c**) and inside view (**d**) of piezoelectric energy-harvesting tile transducer. Each sensor in (**d**) was numbered corresponding to its location designed in (**b**).

The symmetrical placement of the piezoelectric sensors was designed to maximize the induced force applied to any location on the surface of the tile. Four springs with a height of 60 mm, a diameter of 48 mm, and a constant of 1.32 kgf/mm were mounted at the four corners of the frame. The designed tile had a maximum displacement of 3 mm. In addition, to more effectively protect the sensor, indirect touch points were placed on the cover of tile. It should be noted that our energy-harvesting transducer does not pose a risk of destroying the piezoelectric sensor because the impact is transferred to the piezoelectric panel via four indirect touch points and the vertical movement of the cover is limited to 3 mm via the protection springs. For fabrication, the case, frame, and cover were carefully made using a low-cost iron alloy material. Photos of completed piezoelectric energy-harvesting transducer tile are shown in Figure 5.

## 4. Results and Discussion

**Figure 6:**
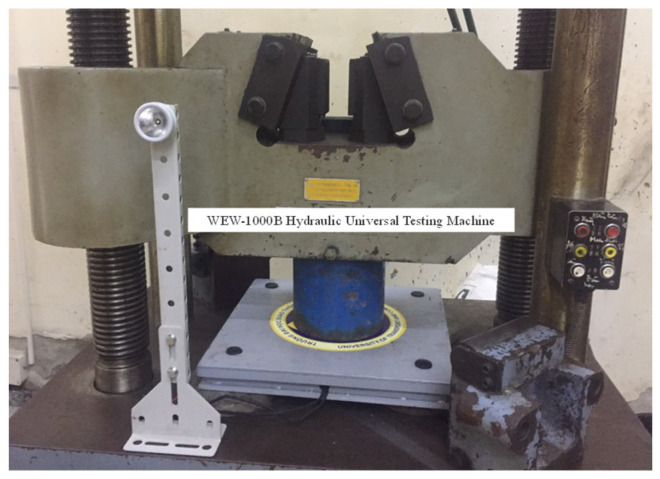
Photo of piezoelectric harvesting transducer tile in universal testing machine.

**Figure 7:**
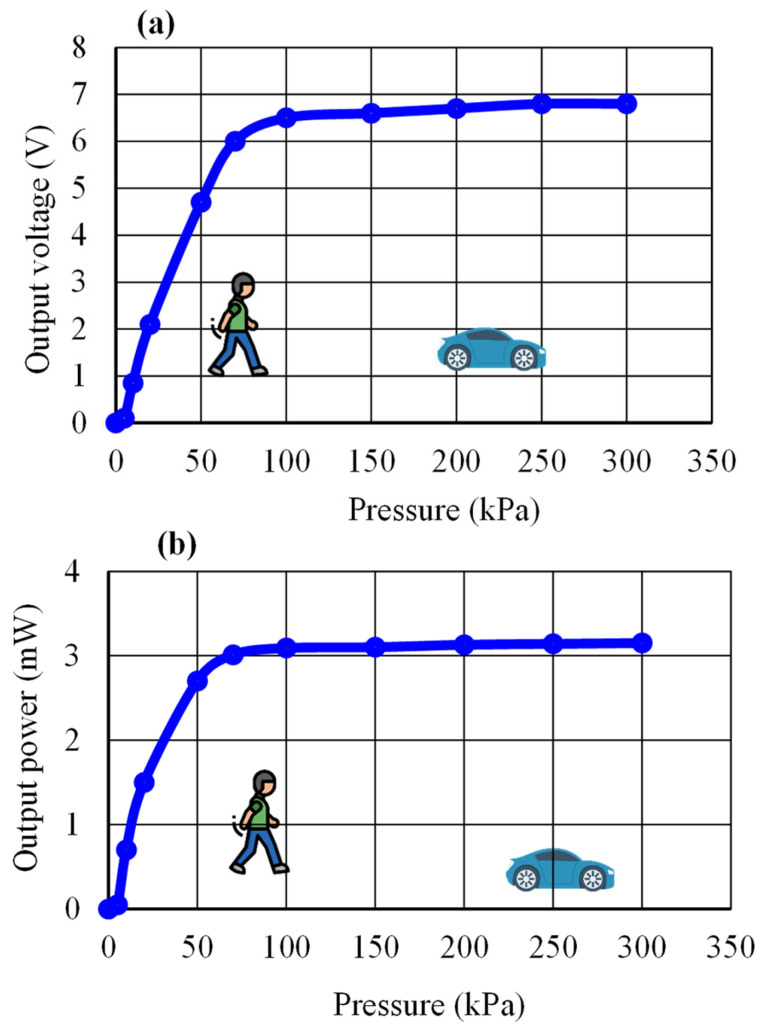
Pressure dependence of (**a**) output voltage and (**b**) output power.

As shown in a photo in Figure 6, the piezoelectric energy-harvesting transducer was placed inside a press machine (WEW-1000B Hydraulic Universal Testing Machine (Chenda tester, Shandong, China) to measure the device’s pressure and displacement dependances. A decade resistance standard resistor (279301, Yokogawa, Musashino City, Japan) was connected to the piezoelectric tile in order to analyze the performance of the energy-harvesting transducer tile at various resistive loads. The electrical output was measured using an oscilloscope (GW INSTEK GDS-1052-U, Taipei, Taiwan). The effects of pressure on the device’s electrical properties at a displacement of 2.5 mm and a load resistance of 15 kΩ are shown in Figure 7. We found that the minimum pressure required to activate the harvesting transducer is 5 kPa. The output voltage and output power increased significantly in the human foot pressure range of 50–100 kPa [15] and were able to withstand a maximum car-on-road pressure of 150 kPa [16], which suggests that the tile device can be used to absorb energy from the movement of a human or a car. 

**Figure 8:**
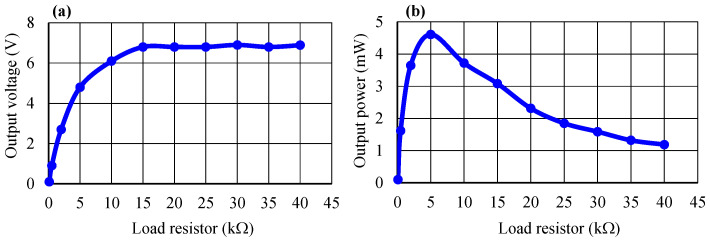
Load resistor dependence of (**a**) output voltage and (**b**) output power.

Figure 8 shows the output voltage and output power of the piezoelectric energy-harvesting transducer as a function of the load resistor at a displacement of 2.5 mm and a pressure of 70 kPa. The output voltage was proportional to the load resistor values, and the maximum output voltage observed was 6.8 V at 15 kΩ. Meanwhile, the output power increased rapidly to a maximum value of 4.8 mW at 5 kΩ and gradually decreased afterward, as shown in Figure 8b. The saturated values of the output voltage and output power are relatively lower than those in the previous works [5,6,7,8] because we only used four sensor panels in the harvesting transducer.

**Figure 9:**
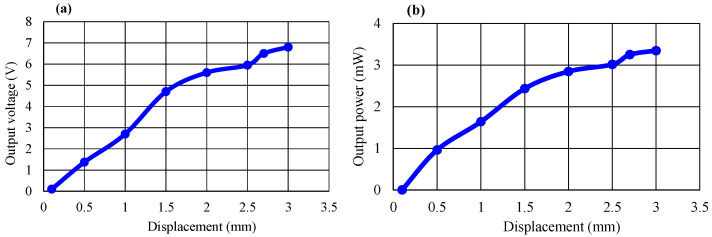
Displacement dependence of (**a**) output voltage and (**b**) output power.

With regard to another important aspect, the displacement dependence of output voltage and output power are shown in Figure 9a,b, respectively. When the displacement parameters were controlled to vary from 1 mm to 3 mm, all measurements were performed at a load resistance of 15 kΩ and a pressure of 70 kPa. As presented in Figure 9, the output voltage or output power values increased with increasing displacement. A previous work [7] indicated that to meet practical operations on the floor or the road, the displacement of the tile should be 2.5 mm. By considering such a recommendation and our experimental data presented in Figure 9, we also selected 2.5 mm as the displacement value of our harvesting transducer tile. 

**Figure 10:**
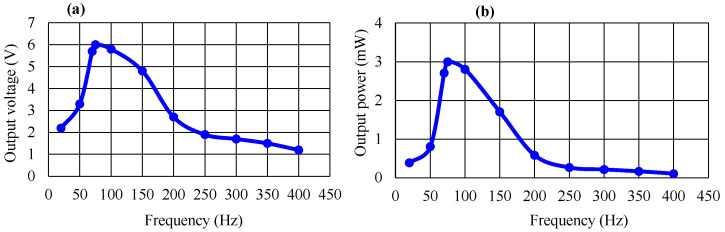
Applied frequency dependence of (**a**) output voltage and (**b**) output power.

Figure 10 shows the dependences of the output voltage and output power of the piezoelectric energy-harvesting transducer on the frequency of the applied force. Experiments were performed at a displacement of 2.5 mm and a pressure of 70 kPa. The output voltage and output power were observed to increase with an increasing frequency, attaining maximum points at 75 Hz and then decreasing gradually. The tendency in Figure 10 is consistent with that in Figure 2b. 

**Figure 11:**
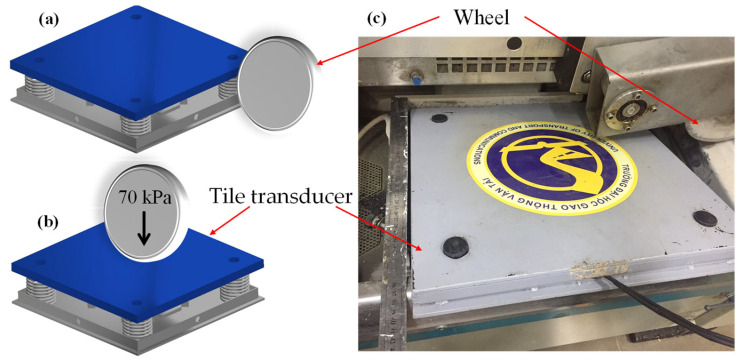
(**a**) Wheel-passing transducer tile. (**b**) Wheel on transducer tile. (**c**) Photo of transducer tile under test with the Wheel-Track Device produced by Hamburg AASHTO T 324.

**Figure 12:**
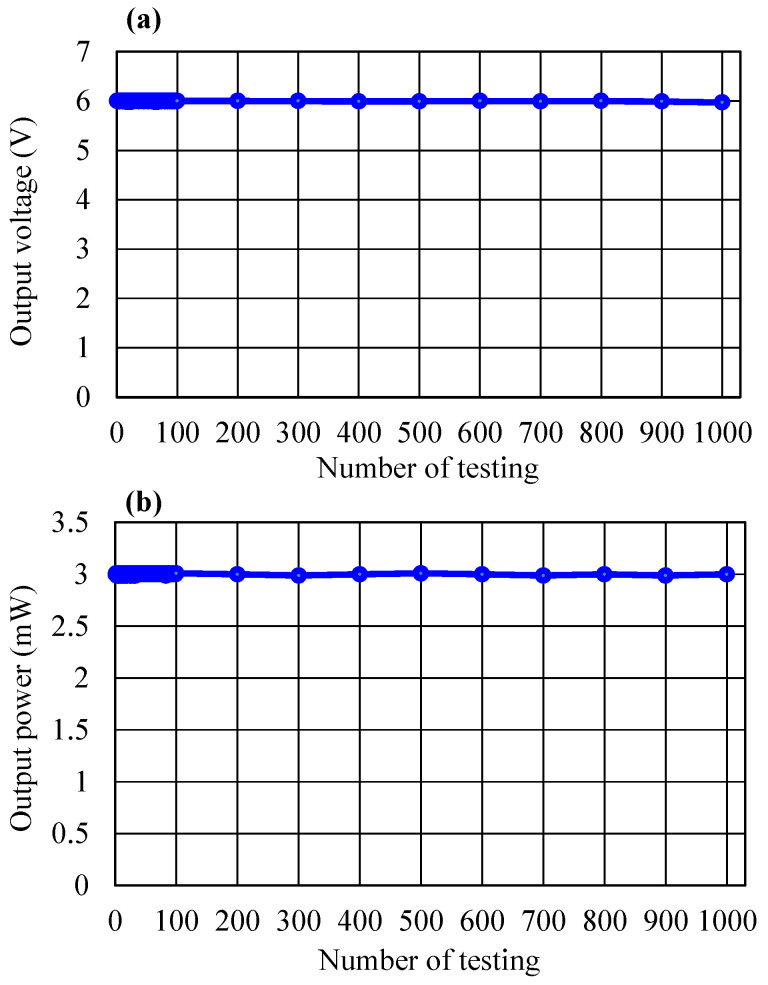
Reliability test of (**a**) output voltage and (**b**) output power.

In order to test the durability of the transducer tile, a Wheel-Track Device produced by Hamburg AASHTO T 324-04 (Infratest, Brackenheim, Germany) with a load pressure of 70 kPa and a frequency of ~75 Hz was used (shown in Figure 11). The output voltage and output power values were repeatedly recorded at a displacement of 2.5 mm and a load resistance of 15 kΩ. As can be seen in Figure 12, the electrical values are almost unchanging after 1000 cycles of testing, suggesting that the energy harvester tile still operated properly without degradation, which is a superior property among the harvesting transducers reported so far [5,6,7,8,9,10,11,12,13,14,17,18]. This achievement was facilitated by the fact that our mechanical design, with indirect touch points and protection springs (Figure 4), suppressed the risk of destroying the piezoelectric sensor during operation and that the flexibility of the sensor allows it to recover its status many times under the influence of an external force.

**Figure 13:**
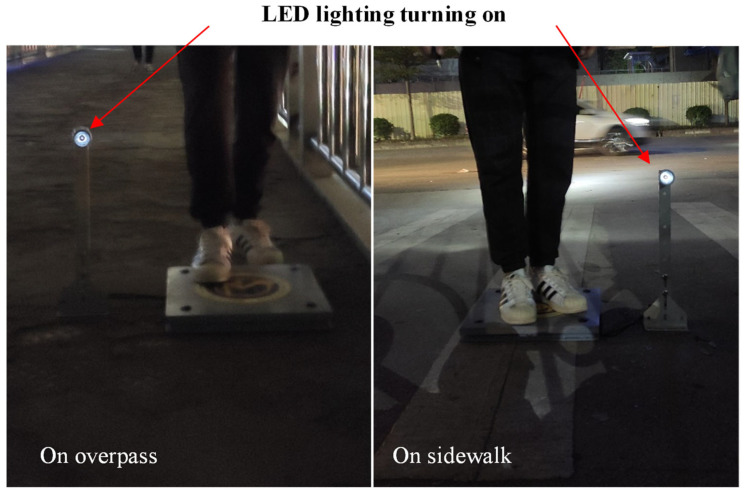
Photos of testing on overpass and sidewalk.

Furthermore, to demonstrate the promising applications of the tile in smart transportation, the harvesting transducer tile was temporarily laid on the floor of an overpass, a sidewalk, and a tunnel walkway (located in Hanoi, Vietnam) to harvest energy from the footsteps of volunteers modelling the behavior pedestrians. Over the course of a few days, several tens of volunteers passed over the tile. For example, as can be seen in Figure 13 and Video S2a–c, when a 68 kg volunteer passed over the energy-harvesting transducer tile, the implemented LED light was powered and illuminated rapidly. Even through the light intensity generated from one tile is not very high, it can be speculated that if the area on the floor was composed of our harvester tiles, the obtained electrical energy may be able to sufficiently illuminate the entire area of the overpass of a walking tunnel or power electronic transportation devices. The remarkable operation test results of the transducer shown in Video S2a–c also confirm the device’s stability. 

## 5. Conclusions

In conclusion, a high-stability energy-harvesting transducer device has been designed and fabricated based on a flexible piezoelectric sensor. The output voltage and output power of the proposed energy-harvesting transducer were investigated as a function of pressure, displacement, and load resistance. The maximum output voltage and maximum output power obtained were 6.8 V and 4.5 mW, respectively, for an input pressure of 150 kN, a displacement of 2.5 mm, and a load resistance of 20 kΩ. Furthermore, the designed structure limits the risk of the piezoelectric sensor being destroyed under the action of an external force. As a result, the energy-harvesting transducer device exhibits high stability at up to 1000 cycles of energy harvesting. Additionally, the piezoelectric device was tested on the floor of an overpass and a walking tunnel. The energy harvested from pedestrian footsteps was enough to power an LED light fixture, which suggests that our tile device has great potential for use in powering the electronic devices in smart transportation. In future research, energy-harvesting transducers with a greater surface area will be developed and evaluated under various operating conditions.

## Figures and Tables

**Table 1:** Frequency dependence of *Z* modulus.

Frequency (Hz)	20	50	70	75	100	150	200	250	300	350	400
*Z* (KΩ)	5.40	24.20	28.00	28.20	27.26	22.00	17.87	12.50	10.00	6.00	2.52

## Data Availability

The data are not publicly available due to confidentiality of institutional data. The data presented in this study are available on request from the corresponding author.

## Supplementary Materials

The following supporting information can be downloaded at: https://www.mdpi.com/article/10.3390/mi14051058/s1. Video S1: Sensor testing via oscilloscope; Video S2a: Testing on overpass. Video S2b: Testing on sidewalk. Video S2c: Testing on tunnel walkway.
